# Improving the Efficiency of Electrocatalysis of Cytochrome P450 3A4 by Modifying the Electrode with Membrane Protein Streptolysin O for Studying the Metabolic Transformations of Drugs

**DOI:** 10.3390/bios13040457

**Published:** 2023-04-04

**Authors:** Polina I. Koroleva, Andrei A. Gilep, Sergey V. Kraevsky, Tatiana V. Tsybruk, Victoria V. Shumyantseva

**Affiliations:** 1 Institute of Biomedical Chemistry, Pogodinskaya Street, 10, Build 8, 119121 Moscow, Russia; 2 Institute of Bioorganic Chemistry of the National Academy of Sciences of Belarus, 220141 Minsk, Belarus; 3 Faculty of Biomedicine, Pirogov Russian National Research Medical University, Ostrovitianov Street, 1, 117997 Moscow, Russia

**Keywords:** cytochrome P450 3A4, electrochemical analysis, streptolysin O, erythromycin, bioreactor, electron transfer, electrocatalysis

## Abstract

In the present work, screen-printed electrodes (SPE) modified with a synthetic surfactant, didodecyldimethylammonium bromide (DDAB) and streptolysin O (SLO) were prepared for cytochrome P450 3A4 (CYP3A4) immobilization, direct non-catalytic and catalytic electrochemistry. The immobilized CYP3A4 demonstrated a pair of redox peaks with a formal potential of −0.325 ± 0.024 V (vs. the Ag/AgCl reference electrode). The electron transfer process showed a surface-controlled mechanism (“protein film voltammetry”) with an electron transfer rate constant (k_s_) of 0.203 ± 0.038 s^−1^. Electrochemical CYP3A4-mediated reaction of N-demethylation of erythromycin was explored with the following parameters: an applied potential of −0.5 V and a duration time of 20 min. The system with DDAB/SLO as the electrode modifier showed conversion of erythromycin with an efficiency higher than the electrode modified with DDAB only. Confining CYP3A4 inside the protein frame of SLO accelerated the enzymatic reaction. The increases in product formation in the reaction of the electrochemical N-demethylation of erythromycin for SPE/DDAB/CYP3A4 and SPE/DDAB/SLO/CYP3A4 were equal to 100 ± 22% and 297 ± 7%, respectively. As revealed by AFM images, the SPE/DDAB/SLO possessed a more developed surface with protein cavities in comparison with SPE/DDAB for the effective immobilization of the CYP3A4 enzyme.

## 1. Introduction

Cytochromes P450 (P450s or CYPs) are a superfamily of enzymes with unique biocatalytic properties. CYPs are common among all classes of living organisms and perform biotransformation of endogenous and exogenous substrates, including pharmacological compounds, toxins and other xenobiotics [1,2,3,4]. The wide variety of CYP isoforms and the types of reactions they catalyze make them promising for use as biocatalysts and bioreactors to study the metabolic transformations of drugs and to obtain pharmacologically significant chemical compounds, including endogenic metabolites, which are difficult to obtain by organic synthesis [5,6,7]. A limitation in the implementation of biocatalytic CYP synthesis is the need to use additional redox partner proteins, such as reductase, cytochrome b5, ferredoxin, flavodoxin and NADPH, as an electron source. In electrochemical systems, the electrode supplies heme iron with reducing equivalents directly from the electrode surface. Using electrochemical methods for the investigation of cytochrome P450 properties is an effective and sensitive way of screening substrates, inhibitors, effectors, activators and metabolites of cytochromes P450 [8,9,10]. For the detection, the substrate-inhibitory potential of this class of hemoproteins and the sophisticated and multistage mechanism of cytochrome P450 electrochemical systems have been studied [11,12,13]. So far, a little-studied area is the operation of cytochromes P450 in the mode of electrochemical bioreactors. This aspect of cytochromes P450 functioning is important for the electro enzymatic production of synthetic drug precursors, structurally complex organic compounds that have optical activity and prodrugs with greater pharmacological activity. In addition, cytochromes P450 are important for the modification and biodegradation of chemical compounds that pollute the environment [2,6,7]. Improvements in cytochrome P450 electrocatalysis remain a significant challenge. Various approaches have been proposed for the creation of electrochemical systems that retain and increase the catalytic activity of the enzymes [6,12,13,14,15,16,17,18,19,20,21,22,23]. Moreover, enzyme immobilization in a volume-confined environment could increase its stability and reduce denaturation and protein unfolding [6]. We have previously reported on the use of an inorganic membrane consisting of anodic aluminum oxide with a highly regular structure and two variants of pore diameters, 0.1 and 0.2 μm, to modify an electrode from a 2D electrode plane to a 3D one for confining cytochrome P450 3A4 (CYP3A4) [20,21]. This approach provided an increase in the efficiency of electrocatalysis of CYP3A4 by a factor of 1.32–2.32, depending on the pore size of the chosen membrane; this result confirms the efficiency of the transition from a 2D to a 3D surface for the immobilization of cytochromes P450 [21].

Human hepatic CYP3A4 is a major enzyme metabolizing a vast variety of marketed drugs with promising synthetic applications [1,24]. Developing methods for investigating enzymes under conditions that mimic and model the natural environment remains a significant challenge. A new direction in this area is the creation of a three-dimensional structure on the electrode surface which would allow the enzyme to be integrated more efficiently and in an orderly manner [19,20,21]. The aim of this work is to develop approaches to improve the efficiency of cytochrome P450 electrocatalysis and the functioning of cytochrome P450 electrodes using natural proteins for obtaining suitable cavities as host frames for enzyme immobilization. From this point of view, we propose an approach that makes it possible to mimic the cellular environment for the creation of a more developed surface with protein cavities for the effective immobilization of the CYP3A4 enzyme based on hybrid biomembranes in the lipid-like bilayer of the electrode modifier didodecyldimethylammonium bromide (DDAB) using the pore-forming protein streptolysin O (SLO) [25,26,27,28,29]. This approach enhances the efficiency of electrochemical CYP3A4-mediated substrate conversion by 297% in the reaction of the N-demethylation of the macrolide antibiotic erythromycin as a well-known CYP3A4 substrate. The novelty of the approach proposed in this work is the enzyme incorporation in the three-dimensional composite DDAB/SLO, leading to an improvement in both the electron transfer properties and the efficiency of CYP3A4 electrocatalysis. The confining effect when CYP3A4 is assembled inside cavities is investigated with direct non-catalytic voltammetry and electroanalysis of the enzymatic reaction, such as the N-demethylation of erythromycin, occurring in the DDAB/SLO composite.

## 2. Materials and Methods

### 2.1. Reagents

In our study, we used human recombinant CYP3A4 (142 µM CYP3A4 in 550 mM potassium phosphate buffer (pH 7.2), containing 0.2% CHAPS, 1 mM dithiothreitol and 20% glycerol (*v*/*v*)). This enzyme was purified according to a published earlier protocol [30] in the Institute of Bioorganic Chemistry (Minsk, Belarus). The concentration of CYP3A4 was determined by a complex formation of a reduced form with carbon monoxide using the absorption coefficient ɛ_450_= 91 mM^−1^ cm^−1^ [31]. Acetylacetone was purchased from Fluka (Switzerland). Potassium hydroxide, potassium dihydrogen phosphate, sodium chloride were purchased from Spectrchem (Moscow, Russia). Acetic acid was purchased from Fisher Scientific (USA). Chemical reagents such as ammonium acetate, erythromycin, chloroform, didodecyldimethylammonium bromide (DDAB), disodium phosphate, DL-dithiothreitol, K_3_[Fe(CN)_6_]/K_4_[Fe(CN)_6_] and streptolysin O from *Streptococcus pyogenes* were purchased from Sigma-Aldrich (St. Louis, MO, USA).

### 2.2. Methods

In this work, screen-printed electrodes, which included a graphite working electrode with a diameter of 0.2 cm, an auxiliary electrode and a silver/silver chloride pseudo reference electrode, were used and obtained from ColorElectronics, Russia (Moscow, Russia, https://www.colorel.ru ( accessed on 25 March 2023)).

To obtain modified electrodes, drop-casting of 1 μL of 0.1 M DDAB in chloroform at the surface of the working electrode was used, then electrodes were left for 10 min until completely dried. For electrode modification with streptolysin O, 1 μL of 4 mg/mL SLO solution in water was mixed with 1 μL 10 mM DL-dithiothreitol in 0.1 M phosphate buffer and incubated for 30 min, then 1 μL of the obtained solution was dropped on the electrodes modified with DDAB. Immobilization of cytochrome P450 3A4 on the electrode surface was carried out as we published earlier [16,20].

### 2.3. Electrochemical Measurements

Potentiostat/galvanostat PGSTAT 302N Autolab (Metrohm Autolab, Utrecht, Netherlands) controlled by NOVA software (version 2.0) was used in all electrochemical measurements (room temperature (23 °C) and in 0.1 M potassium phosphate buffer (pH 7.4) containing 0.05 M NaCl as a supporting electrolyte). All potentials were given relative to the Ag/AgCl reference electrode. Cyclic voltammetry technique under anaerobic Ar-saturated electrolyte buffer was employed. Electrocatalytic measurements in the presence of substrate under aerobic conditions were performed at room temperature in an air-saturated buffer [16,20]. Electrolysis was performed at the controlled potential of E = −0.5 V for 20 min.

Erythromycin N-demethylation was registered based on formaldehyde formation as described [32,33,34]. Electrochemical parameters were means ± standard deviations from three electrodes.

### 2.4. AFM Measurements

AFM measurements were recorded in the “Human Proteom Core Facility” using a Dimension FastScan microscope (Bruker, Billerica, MA, USA) equipped with commercial Fastscan-A cantilevers, in tapping mode, in air.

Square plates (approximately 7 mm × 7 mm) from HOPG were used as substrates in AFM experiments. A 2 μL DDAB solution was deposited directly onto a freshly cleaved HOPG. Then, either the sample was left for 15 s for adsorption or a 0.5 μL SLO solution was added, and the sample was left for 5 min, gently rinsed from the pipet with 500 μL of Milli-Q water and dried in nitrogen flow. Image processing was performed using FemtoScan software [35].

## 3. Results and Discussion

### 3.1. Characterization of the Composite by AFM

**Figure 1:**
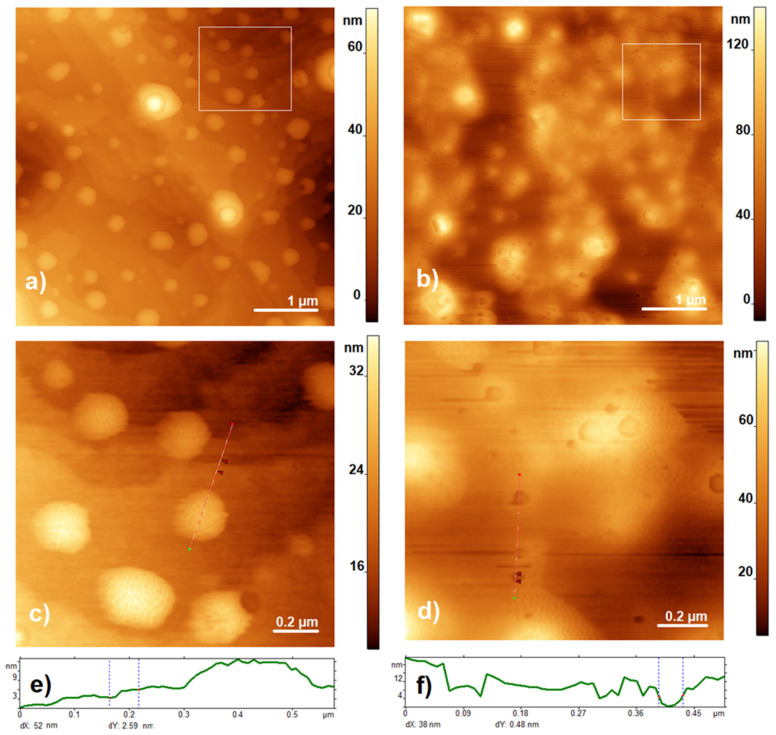
AFM images of DDAB (left column) and DDAB/SLO (right column). (**a**,**b**) Tapping mode height AFM images, (**c**,**d**) zoomed-in regions, shown by the white squares and (**e**,**f**) profiles along dotted lines with dX and dY measured between two red arrowhead points.

The composite of DDAB/SLO was characterized by AFM images. As shown in Figure 1a,b, SLO incorporated in lipid-like biomembranes displayed protein cavities with irregularly formed structures.

AFM measurements were used to compare the surface of DDAB layers with the DDAB/SLO composite, as shown in Figure 1. Our values for the DDAB bilayer thickness measured in air are in good agreement with the values of ~2–3 nm reported by Li et al. (Figure 3c in [36]) but slightly lower than the thickness of the fully hydrated DDAB bilayer with a value of 3.2 nm measured in solution and reported in [37]. The specimen with the addition of SLO showed large surface roughness and the formation of a significant number of cavities of a broad range of diameters 10–100 nm in lipid-like biomembranes (Figure 1b,d), wherein the depth of the formed cavities was more than 4 nm, i.e., which is more than the bilayer thickness.

### 3.2. Comparative Characterization of the Electrochemical System SPE/DDAB/CYP3A4 and SPE/DDAB/SLO/CYP3A4

The voltammetric behavior of the modified SPEs (SPE/DDAB and SPE/DDAB/SLO) was studied by cyclic voltammetry using well-known electroactive redox probe K_3_[Fe(CN)_6_]/K_4_[Fe(CN)_6_] (Figure S1). The values of E_red_, E_ox_, ΔE, E_1/2_, I_red_ and I_ox_ were determined at a scan rate of 0.05 V/s (Table S1). Cyclic voltammetry of SPE/DDAB and SPE/DDAB/SLO both show well-defined reversible redox peaks with a peak-to-peak separation ΔEp of 0.123 ± 0.008 V in the case of SPE/DDAB/SLO. This implies that the more reversible redox performance of [Fe(CN)_6_]^3−/4−^ occurs when the SLO protein was used for additional SPE/DDAB modification.

The Randles–Sevcik equation was used for the calculation of the electroactive surface area, A (cm^2^), of the electrode [38,39,40]:

(1) $$\mathrm{Ip}={2.69\times 10}^{5}\times A\times \sqrt{D}\times \sqrt{\nu }\times C\times n^{3/2}$$

where A is the electroactive surface area in cm^2^, D is the diffusion coefficient in cm^2^/s, C is the concentration in mol/cm^3^, ν is the scan rate in V/s and n is the number of electrons transferred in the redox cycle. Ip is the current maximum in amps. By substituting the known values of D = 7.6 × 10^−6^ cm^2^/s [41,42], n = 1 and C = 0.005 M for the used K_3_[Fe(CN)_6_]/K_4_[Fe(CN)_6_] redox probe into Equation (1), the surface areas of each electrode modification can easily be extracted from the slope with the dependence of Ip vs. ν^1/2^. The calculated values of A are also summarized in Table S1.

Protein immobilization on the surface of the working electrode provided efficient electron transport. Nevertheless, protein interaction with a plane electrode surface could cause protein denaturation and the missing or reduced catalytic properties of the enzyme [43]. Mammalian cytochromes P450 are membrane proteins; therefore, many approaches for the study of these hemeproteins use membrane-like substances to immobilize cytochromes P450. Different modification strategies, including membrane-like surfactants, are helpful decisions working with “solid” electrodes. Such an approach for immobilization support both effective electron transfer and the maintenance of the tertiary structure of the protein. Electrodes modified by means of the synthetic lipid-like compound didodecyldimethylammonium bromide as a planar (2D) surface are widely used for the investigation of direct and catalytic electrochemistry of hemeproteins and, especially, CYP enzymes [44,45,46].

**Scheme 1:**
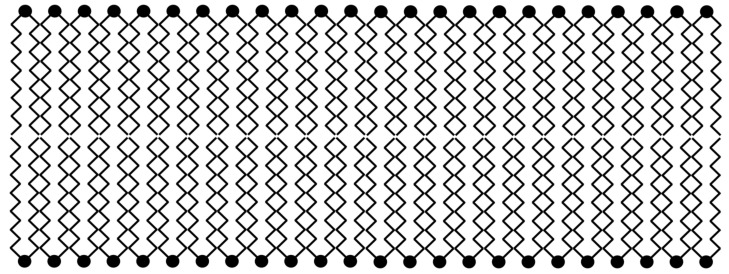
Schematic presentation of synthetic lipid-like compound didodecyldimethylammonium bromide (DDAB) [CH_3_(CH_2_)_11_]_2_N^+^(CH_3_)_2_(Br^−^) bilayer.

The membrane-like substance DDAB forms a bilayer (Scheme 1), which is similar, in part, to a cell membrane; such a structure is described in [44].

Earlier, the electrochemical properties of the hemeprotein CYP3A4 immobilized on the screen-printed graphite electrode modified with the synthetic lipid-like compound DDAB were investigated, in detail, in both anaerobic and aerobic conditions [16,17,18]. For the enhancement in catalytic activity and surface coverage of the electrode with the hemeprotein, we propose the use of a composite of DDAB and the pore-forming bacterial toxin streptolysin O (SLO). SLO is a glycosylated protein derived from the gram-positive microorganism *Streptococcus pyogenes*, with an approximate molecular weight of 61.5 kDa, as published in [19,25,26,27]. In this case, the transition from a 2D lipid bilayer to a 3D type of electrode demonstrates a more developed surface with protein cavities for effective immobilization of the CYP3A4 enzyme.

**Figure 2:**
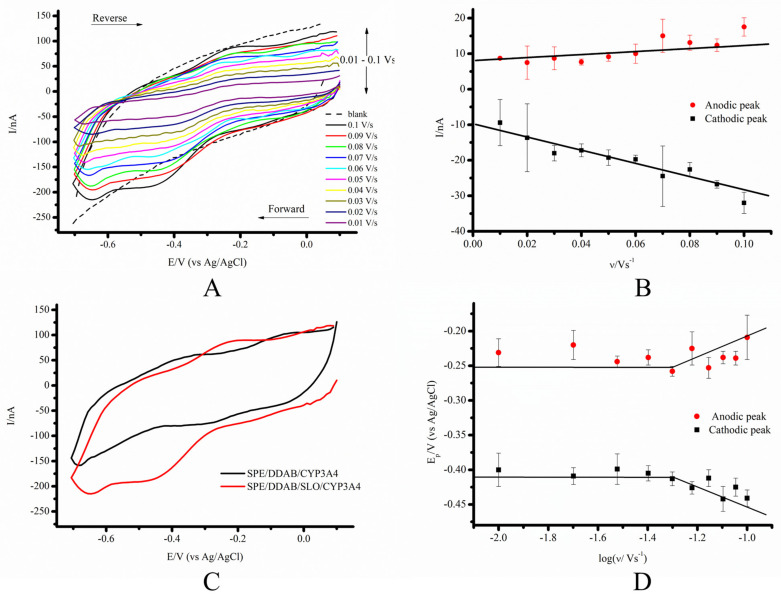
(**A**) CVs of SPE/DDAB/SLO/CYP3A4 in anaerobic conditions. (**B**) Scan rate dependence of the cathodic and anodic peak currents on the potential for SPE/DDAB/SLO/CYP3A4. (**C**) CVs of SPE/DDAB/CYP3A4 and SPE/DDAB/SLO/CYP3A4 at scan rate of 0.1 V/s. (**D**) Trumpet plots displaying the oxidation and reduction peak potentials with logarithm scan rate for SPE/DDAB/SLO/CYP3A4. Measurement parameters: potential sweep rate from 0.01 to 0.1 V/s, potential range from −0.7 to 0.1 V (vs. Ag/AgCl). The measurements were carried out in 1 mL of supporting electrolyte 0.1 M potassium phosphate buffer (pH 7.4) containing 0.05 M NaCl.

For the investigation of the electrochemical characteristics of the system based on CYP3A4 and graphite electrodes modified with the synthetic membrane-like compound DDAB and the membrane protein SLO (SPE/DDAB/SLO), we employed the cyclic voltammetry technique under both an anaerobic and aerobic electrolyte buffer. Cyclic voltammograms (CVs) of CYP3A4 at scan rates of 0.01–0.1 V/s (Figure 2A) demonstrated the reversible redox process CYP-Fe^III^ + ē (from the electrode) ⇄ CYP-Fe^II^ occurring in the heme redox center, where CYP-Fe^III^ is the oxidized (ferri) form, and CYP-Fe^II^ is the reduced (ferro) form of CYP3A4. We observed surface-controlled processes named “protein film voltammetry” and not diffusion-controlled processes, which is confirmed by the linear dependence of the maximum amplitudes of the cathodic and anodic currents on the scan rates [47,48] (Figure 2B). The midpoint potentials E^0′^ of CYP3A4 for both types of electrodes were calculated using the equation E^0′^ = (E_pc_ + E_pa_)/2 and demonstrated very close values, corresponding to −0.302 ± 0.010 V and −0.325 ± 0.024 V for the DDAB and DDAB/SLO films, respectively. The DDAB/SLO composite revealed a negative shift in the formal potential of CYP3A4 in comparison with SPE/DDAB. As can be seen from Figure 2C, SPE/DDAB/SLO/CYP3A4 demonstrated a more pronounced CV in comparison with DDAB modification. In order to confirm the surface-controlled regime, the peak potential of the oxidation and reduction peaks were plotted against the logarithm scan rate (the so-called “trumpet plot”) (Figure 2D).

The heterogeneous electron transfer constants (k_s_) at the scan rate 0.1 mV s^−1^ were determined according to Laviron’s model using Equation (2) [49]:

(2) $${\mathrm{Ink}}_{s}=\alpha \ln\left(1-\alpha \right)+\left(1-\alpha \right)\ln\alpha -\ln\left(\frac{\mathrm{RT}}{\mathrm{nF}\nu }\right)-\alpha \left(1-\alpha \right)\left(\frac{\mathrm{nF}∆E_{p}}{\mathrm{RT}}\right)$$

where k_s_ is the heterogeneous electron transfer constant, s^−1^; ν is the scan rate, V s^−1^; α is the transfer coefficient, assumed as 0.5; R is 8.314 J mol^−1^ K^−1^; T is the temperature, K; and ∆E_p_ is the peak separation between anodic and cathodic peak potentials, V (ΔE > 0.2 V).

The heterogeneous electron transfer rate constant k_s_ for SPE/DDAB/SLO/CYP3A4 was 0.203 ± 0.038 s^−1^, and two times lower than in the case of SPE/DDAB/CYP3A4 (0.51 ± 0.03 s^−1^). However, these parameters are comparable to earlier published data on a Au/CYP3A4 electrode, corresponding to values from 0.6 to 3.7 s^−1^ [50].

The surface coverage of the electroactive protein was calculated according to Faraday’s law by Equation (3) [48]:

(3) $$\Gamma_{0}=\frac{Q}{\mathrm{nFA}}$$

where F is the Faraday constant, 96,485 C mol^−1^; A is the surface area of the working electrode, cm^−2^; Q is the electric charge calculated from integration of voltammogram peaks, C; n is the number of transferred electrons (n = 1 for cytochrome P450s in accordance with scheme Fe^III^ + ē ⇄ Fe^II^) [1,2,3,4]; and Γ_0_ is the surface coverage or surface concentration of the electroactive protein, mol cm^−2^ [48]. However, all the electroanalytical parameters of SPE/DDAB/SLO/CYP3A4 were comparable with SPE/DDAB/CYP3A4; these values permit us to use an additional modification of the lipid-like surface of SPE with pore-forming SLO for investigation of electrocatalytic properties of CYP3A4 using a new type of modifier.

The electrochemical parameters calculated from the experimentally obtained data for SPE/DDAB/SLO/CYP3A4 in comparison with SPE/DDAB/CYP3A4 in the argon-saturated electrolyte buffer are summarized in Table 1.

**Figure 3:**
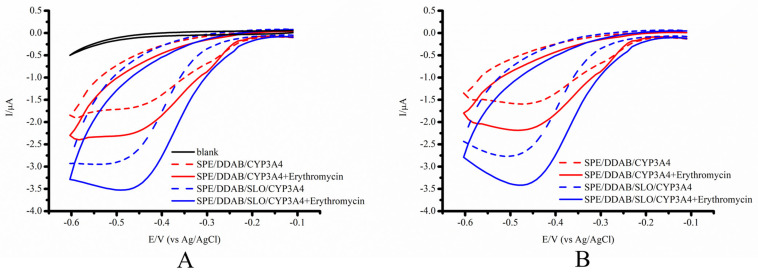
(**A**) CVs of SPE/DDAB, SPE/DDAB/CYP3A4, SPE/DDAB/CYP3A4 in the presence of substrate erythromycin as 100 µM; SPE/DDAB/SLO/CYP3A4, SPE/DDAB/SLO/CYP3A4 in the presence of substrate erythromycin as 100 µM at a scan rate of 0.1 V/s. (**B**) CVs of SPE/DDAB/CYP3A4, SPE/DDAB/CYP3A4 with 100 µM erythromycin, SPE/DDAB/SLO/CYP3A4, SPE/DDAB/SLO/CYP3A4 with 100 µM erythromycin, at a scan rate of 0.1 V/s with the background subtracted curves.

In an oxygen-containing supporting electrolyte, the reduced Fe^II^ ions actively bind with oxygen as a co-substrate, and only the cathodic reduction peak is recorded in accordance with the irreversible well-known reaction Fe^II^ + O_2_ → Fe^II^ O_2_, which increases with an increase in the scan rate. The reduction potentials of SPE/DDAB/SLO/CYP3A4 in an aerobic buffer are characterized by a slight shift to the negative cathodic potential compared to SPE/DDAB/CYP3A4 with ΔE~0.02 V (Table 2 and Figure 3A,B).

An efficient electrochemical system based on DDAB/SLO modification with a more developed surface with additional protein cavities made it possible to further study the electrocatalytic properties of SPE/DDAB/SLO/CYP3A4 for the development of bioreactors based on the cytochrome P450 family.

We have studied the electrochemical system SPE/DDAB/SLO/CYP3A4 in the presence of the substrate erythromycin as a marker substrate of this form of the CYP enzyme [1,3,35,51]. Erythromycin is an antibiotic from the macrolide group, and it possesses broad pharmacological action against both gram-positive and gram-negative bacteria (such as *Staphylococcus aureus*, *Streptococcus pneumoniae*, *Streptococcus pyogenes*, *Neisseria gonorrhoeae*, *Haemophilus influenzae* and *Bordetella pertussis*) [52,53].

CYPs are categorized as bi-substrate enzymes with the participation of two substrates, such as oxygen and an organic compound [1,2,3,4,49]. In the presence of oxygen, CYP3A4 effectively catalyzed the N-demethylation of erythromycin [32,33]. The analytical sensitivity of SPE/DDAB/CYP3A4 and SPE/DDAB/SLO/CYP3A4 for erythromycin is 193 ± 21 and 389 ± 45 nA µM^−1^ cm^−1^, respectively. The potentials for the start of catalysis of erythromycin (E_onset_) in the electrochemical system SPE/DDAB/CYP3A4 and SPE/DDAB/SLO/CYP3A4 are −0.228 ± 0.003 V and −0.264 ± 0.020 V, respectively.

As can be seen from Figure 3 and Table 2, SPE/DDAB/SLO/CYP3A4 possesses a more pronounced catalytic current, registered in the presence of oxygen and erythromycin.

Electrochemical CYP3A4-mediated reaction of N-demethylation of erythromycin was explored with the following parameters: an applied potential of −0.5 V and a duration time of 20 min. The formaldehyde concentration was measured spectrophotometrically at 412 nm using Nash reagent (100 mM acetic acid, 4M ammonium acetate and 40 mM acetylacetone) [23,24,25]. The system with SLO as an electrode modifier showed conversion of erythromycin registered by means of formaldehyde appearance with an efficiency higher than the electrode modified with DDAB only. Based on the measured concentrations of released formaldehyde in the reaction of CYP3A4-electrocatalytic N-demethylation of erythromycin with SPE/DDAB/CYP3A4 and SPE/DDAB/SLO/CYP3A4, the relative activities were equal to 100 ± 22% and 297 ± 7% with V_max_ 1.52 ± 0.34 × 10^−10^ and 4.52 ± 0.33 × 10^−10^ M min^−1^, respectively (Table 3).

**Figure 4:**
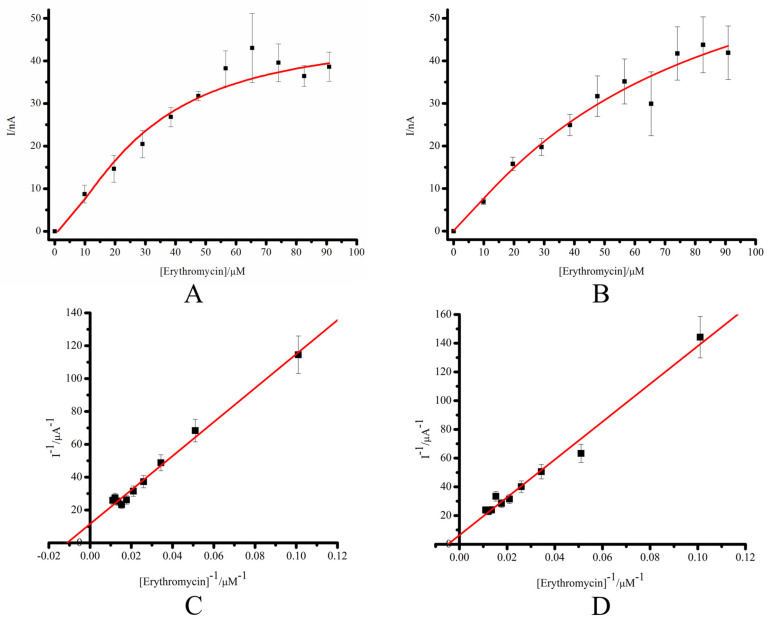
Michaelis–Menten plot of the dependence of the catalytic current of SPE/DDAB/CYP3A4 (**A**) and SPE/DDAB/SLO/CYP3A4 (**C**) on erythromycin concentration 10–100 μM; (**B**) the Lineweaver–Burk plot for SPE/DDAB/CYP3A4 and SPE/DDAB/SLO/CYP3A4 (**D**). Titration of CYP3A4 was performed with 10 mM erythromycin dissolved in ethanol. The ethanol concentration in the cell did not exceed 1% by volume. The measurements were carried out in 1 mL of potassium phosphate buffer at a fixed working electrode potential of −0.5 V (vs. Ag/AgCl). The values are the means from at least 3 experiments ± S.D.

To assess the comparable kinetic parameters of the electrochemical systems, we performed chronoamperometric titration of CYP3A4 immobilized on SPE/DDAB or SPE/DDAB/SLO with erythromycin. The apparent Michaelis constants, K_M_, were calculated from electrochemical data using the Michaelis–Menten equation and its electrochemical form (Equations (4) and (5)). The Michaelis–Menten plots of the dependence of the catalytic current of SPE/DDAB/CYP3A4 and SPE/DDAB/SLO/CYP3A4 electrodes for experimental amperometric titration of erythromycin are shown in Figure 4A–D and Table 3. Based on the results of titration in double reciprocal coordinates (Lineweaver–Burk diagram in 1/V, 1/[S]), the values of the Michaelis constants, K_M_, were calculated [12,33,34,54].

(4) $$I_{\mathrm{cat}}=\frac{I_{\mathrm{cat}\;\max}[S]}{{K_{M}}^{\mathrm{app}}+[S]}$$

(5) $$I_{\mathrm{cat}}=\frac{\mathrm{nFA}\Gamma_{0}k_{\mathrm{cat}}[S]}{{K_{M}}^{\mathrm{app}}+[S]}$$

where I_cat_ is the catalytic current at an appropriate substrate concentration, A; I_cat max_ is the maximum catalytic current at full saturation of the enzyme, A; [S] is the substrate concentration, M; and K_M_^app^ is the electrochemical Michaelis constant, M.

The Michaelis constants, K_M_, of erythromycin for CYP3A4 immobilized on SPE/DDAB or SPE/DDAB/SLO/CYP3A4 were determined as 8.98 ± 1.2 × 10^−5^ M and 20.7 ± 2.5 × 10^−5^ M, respectively. Our results are in line with and in the same order of magnitude as previously published data (Table S2) [34,54]. The reason for the decrease in K_M_ for the SPE/DDAB/SLO/CYP3A4 system may be related to a decrease in the diffusion of the substrate to the enzyme located in the cavities of SLO compared to the enzyme located in the lipid-like DDAB surface of the electrode.

## 4. Conclusions

We studied the direct electron transfer of human CYP3A4 using screen-printed electrodes (SPEs) modified with didodecyldimethylammonium bromide (DDAB) as a membrane-like synthetic surfactant and streptolysin O (SLO). We showed that SLO on the surface of lipid-like DDAB forms a highly developed surface with cavities, which permit the immobilization of the CYP3A4 enzyme for direct non-catalytic and catalytic electrochemistry. The immobilized CYP3A4 demonstrated a pair of redox peaks with a formal potential of −0.325 ± 0.024 V. For the substrate conversion of erythromycin as an N-demethylation reaction, an applied potential of E = −0.5 V was used. The system with DDAB/SLO as the electrode modifier was suitable for analyzing the electrocatalysis. The efficiencies of electro enzymatic erythromycin N-demethylation in SPE/DDAB/CYP3A4 and SPE/DDAB/SLO/CYP3A4 were equal to 100 ± 22% and 297 ± 7%, respectively. AFM analysis of electrodes modified with streptolysin O (SPE/DDAB/SLO) revealed a more developed surface with protein cavities for effective immobilization of the CYP3A4 enzyme.

## Figures and Tables

**Table 1:** Electroanalytical parameters of CYP3A4 immobilized on SPE/DDAB and SPE/DDAB/SLO under anaerobic conditions.

Electrode	E_c_, V	E_a_, V	E^0^′, V	ΔE, V	k_s_, s^−1^	Γ_0_, mol/cm^2^
SPE/DDAB/ CYP3A4 [14]	−0.377 ± 0.008	−0.227 ± 0.010	−0.302 ± 0.010	−0.150 ± 0.009	0.51 ± 0.030	2.7 ± 0.2 ×10^−11^
SPE/DDAB/SLO/ CYP3A4	−0.441 ± 0.016	−0.209 ± 0.032	−0.325 ± 0.024	−0.230 ± 0.044	0.203 ± 0.038	1.75 ± 0.26 × 10^−11^

Data are calculated based on CV at scan rate of 0.1 V/s.

**Table 2:** Electrocatalytic parameters of CYP3A4 immobilized on SPE/DDAB and SPE/DDAB/SLO under aerobic conditions. The parameters were calculated at a scan rate of 0.1 V/s, and the concentration of erythromycin is 100 μM.

Electrode	E_red_, V	E_cat_, V	E_onset_, V	I_O2_, A × 10^−7^	I_Er_, A × 10^−7^	I_Er_/I_O2_	Sensitivity, nA µM^−1^ cm^−1^
SPE/DDAB/ CYP3A4	−0.438 ± 0.006	−0.438 ± 0.006	−0.228 ± 0.003	−3.71 ± 0.51	−6.05 ± 0.63	1.63 ± 0.14	193 ± 21
SPE/DDAB/ SLO/CYP3A4	−0.462 ± 0.001	−0.445 ± 0.006	−0.264 ± 0.020	−9.01 ± 1.59	−12.22 ± 2.49	1.57 ± 0.17	389 ± 45

I_O2_ is a reduction current in aerobic conditions; I_Er_ is a current in the presence of 100 μM erythromycin as a substrate of CYP3A4 (co-called catalytic current); E_red_ is a reduction potential in the presence of oxygen; E_cat_ is a reduction potential in the presence of oxygen and 100 μM erythromycin; E_onset_ is the potential for the start of catalysis of 100 μM erythromycin. The analytical sensitivity was calculated as the ratio of maximum amplitude of catalytic current to the appropriate erythromycin concentration.

**Table 3:** Efficiency of erythromycin electrocatalytic CYP3A4-dependent N-demethylation at the defined potential of −0.5 V for a duration of 20 min.

Electrode	I_cat max_, A	K_M_, M	V_max_, M·min^−1^
SPE/DDAB/CYP3A4	8.69 ± 0.94 × 10^−8^	8.98 ± 1.2 × 10^−5^	1.52 ± 0.34 × 10^−10^
SPE/DDAB/SLO/CYP3A4	15.8 ± 1.7 × 10^−8^	20.7 ± 2.5 × 10^−5^	4.52 ± 0.33 × 10^−10^

## Data Availability

Not applicable.

## Supplementary Materials

The following supporting information can be downloaded at: https://www.mdpi.com/article/10.3390/bios13040457/s1, Figure S1: Cyclic voltammograms of 5 mM of K_3_[Fe(CN)_6_] on SPE/DDAB (black line) and SPE/DDAB/SLO (red line). The measurements were carried out in 5 mM of K_3_[Fe(CN)_6_] at ambient temperature in potential range from −0.3 mV to +0.8 V (vs Ag/AgCl) at scan rates of 0.05 V/s; Table S1: Electrochemical parameters of SPE/DDAB and SPE/DDAB/SLO in electroactive redox probe 5 mM K_3_[Fe(CN)_6_]/K_4_[Fe(CN)_6_]; Table S2: Comparison of the Michaelis constants KM of erythromycin for CYP3A4 in electrochemical and microsomal systems.
