# Supplementary material for: Improving the Efficiency of Electrocatalysis of Cytochrome P450 3A4 by Modifying the Electrode with Membrane Protein Streptolysin O for Studying the Metabolic Transformations of Drugs
Source: Biosensors (Basel). 2023 Apr 4;13(4):457. doi: 10.3390/bios13040457 (PMC10136652; doi:10.3390/bios13040457)
Supplement: Supplementary file 1 [file biosensors-13-00457-s001.zip › biosensors-2248420-supplementary.pdf]

Supplemental information

Improving the Efficiency of Electrocatalysis of Cytochrome P450 3A4 by Modifying the Electrode with Membrane Protein Streptolysin O for Studying the Metabolic Transformations of Drugs

Polina I. Koroleva[1], Andrei A. Gilep[1,2], Sergey V. Kraevsky[1], Tatiana V. Tsybruk[2], Victoria V. Shumyantseva[1,3*]

[1] Institute of Biomedical Chemistry, Pogodinskaya Street, 10, Build 8, 119121 Moscow, Russia;

11126699@mail.ru (P.I.K.); andrei.gilep@gmail.com (A.A.G.); skraevsky@mail.ru (S.V.K)

[2] Institute of Bioorganic Chemistry of the National Academy of Sciences of Belarus, 220141 Minsk, Bela-rus; tvshkel@gmail.com (T.V.T)

[3]Faculty of Biomedicine, Pirogov Russian National Research Medical University, Ostrovitianov Street, 1, 117997 Moscow, Russia

[*] Correspondence: viktoria.shumyantseva@ibmc.msk.ru

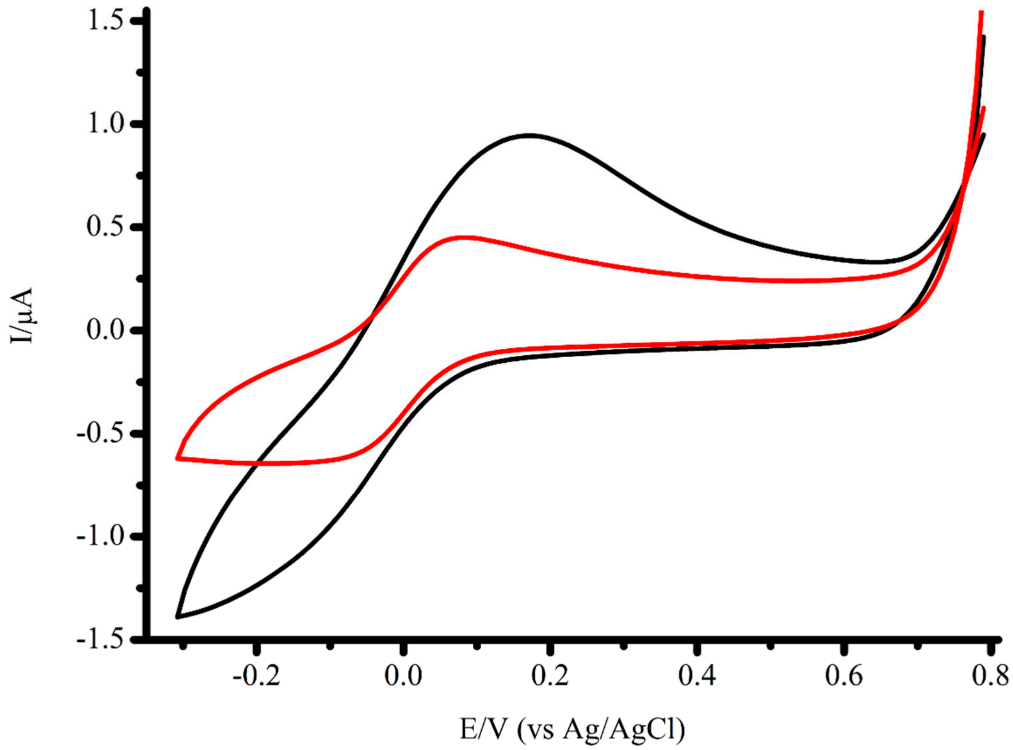

Figure S1. Cyclic voltammograms of 5 mM of $K_3[Fe(CN)_6]$ on SPE/DDAB (black line) and SPE/DDAB/SLO (red line). The measurements were carried out in 5 mM of $K_3[Fe(CN)_6]$ at ambient temperature in potential range from -0.3 mV to +0.8 V (vs Ag/AgCl) at scan rates of 0.05 V/s.

Table S1. Electrochemical parameters of SPE/DDAB and SPE/DDAB/SLO in electroactive redox probe 5 mM $K_3[Fe(CN)_6]/K_4[Fe(CN)_6]$.

	Electrode	
	**SPE/DDAB**	**SPE/DDAB/SLO**
E_{Red}, V	-0.136 ± 0.011	-0.078 ± 0.008
E_{Ox}, V	0.108 ± 0.006	0.045 ± 0.01
ΔE, V	0.245 ± 0.016	0.123 ± 0.008
$E_{1/2}$, V (vs Ag/AgCl)	-0.014 ± 0.003	-0.016 ± 0.008
I_{Red},A×10^{-7}	-2 ± 0.7	-1.3 ± 0.4
I_{Ox}, A×10^{-7}	5.5± 0.41	1.2 ± 0.5
**Electroactive surface area, A, cm^2**	0.000092	0.00011

Table S2. Comparison of the Michaelis constants K_M of erythromycin for CYP3A4 in electrochemical and microsomal systems.

System	Km, M	Reference
GC/PDDA/CYP3A4	86 ± 3×10^{-6}	[34]
SPE/DDAB/ CYP3A4	3.4 ± 0.3×10^{-6}	[33]
SPE/DDAB/CYP3A4	89.8±12×10^{-6}	This work
HLM CYP3A4 (Human liver microsomes)	88×10^{-6}	[54]
Expressed CYP3A4	33×10^{-6}	[54]
